# Changes of immune microenvironment in head and neck squamous cell carcinoma in 3D-4-culture compared to 2D-4-culture

**DOI:** 10.1186/s12967-023-04650-1

**Published:** 2023-10-31

**Authors:** Jian Xiao, Yexun Song, Ru Gao, Mingyang You, Changxin Deng, Guolin Tan, Wei Li

**Affiliations:** Department of Otolaryngology-Head and Neck Surgery, The Third Xiangya Hospital of Central South University, Changsha, 410013 China

**Keywords:** Head and neck squamous cell carcinoma, Immune microenvironment, 2D-4-culture, 3D-4-culture

## Abstract

**Background:**

The immune system plays a crucial role in initiating, progressing, and disseminating HNSCC. This study aims to investigate the differences in immune microenvironments between 2D-4-culture and 3D-4-culture models of head and neck squamous cell carcinoma (HNSCC) cells (FaDu), human fibroblasts (HF), human monocytes (THP-1), and human endothelial cells (HUVEC).

**Methods:**

For the 3D-4-culture model, FaDu:HF:THP-1 (2:1:1) were inoculated in an ultra-low attachment culture plate, while HUVECs were placed in a transwell chamber. The ordinary culture plate was used for the 2D-4-culture model. Tumor-associated macrophage markers (CD163), tumor-associated fibroblast markers (FAP), and epithelial-mesenchymal transition (EMT) were detected by western blot. Inflammatory cytokines (IL-4, IL-2, CXCL 10, IL-1 β, TNF-α, CCL 2, IL-17 A, IL-6, IL-10, IFN-γ, IL-12 p 70, CXCL 8, TGFβ1) in the supernatant were measured by flow cytometry. HUVEC migration was observed under a microscope. The 3D spheres were stained and observed with a confocal microscope. CCK8 assay was used to detect the resistance of mixed cells to cisplatin in both 2D-4-culture and 3D-4-culture.

**Results:**

After three days of co-culture, the 3D-4-culture model showed increased expression levels of CD163 and FAP proteins (both P < 0.001), increased expression of E-cadherin protein and N-cadherin protein expression (P < 0.001), decreased expression of vimentin (P < 0.01) and Twist protein (P < 0.001). HUVEC migration significantly increased (P < 0.001), as did the concentrations of IP-10, MCP-1, IL-6, and IL-8 (all P < 0.001). Confocal microscopy showed that 3D-4-culture formed loose cell clusters on day 1, which gradually became a dense sphere surrounded by FaDu cells invading the inside. After co-culturing for 24 h, 48 h, and 72 h, the resistance of mix cells to cisplatin in 3D-4-culture was significantly higher than in 2D-4-culture (P < 0.01 for all).

**Conclusion:**

Compared to 2D-4-culture, 3D-4-culture better simulates the in vivo immune microenvironment of HNSCC by promoting fibroblast transformation into tumor-associated fibroblasts, monocyte transformation into tumor-associated macrophages, enhancing endothelial cell migration ability, partial EMT formation in HNSCC cells, and is more suitable for studying the immunosuppressive microenvironment of HNSCC.

**Graphical abstract:**

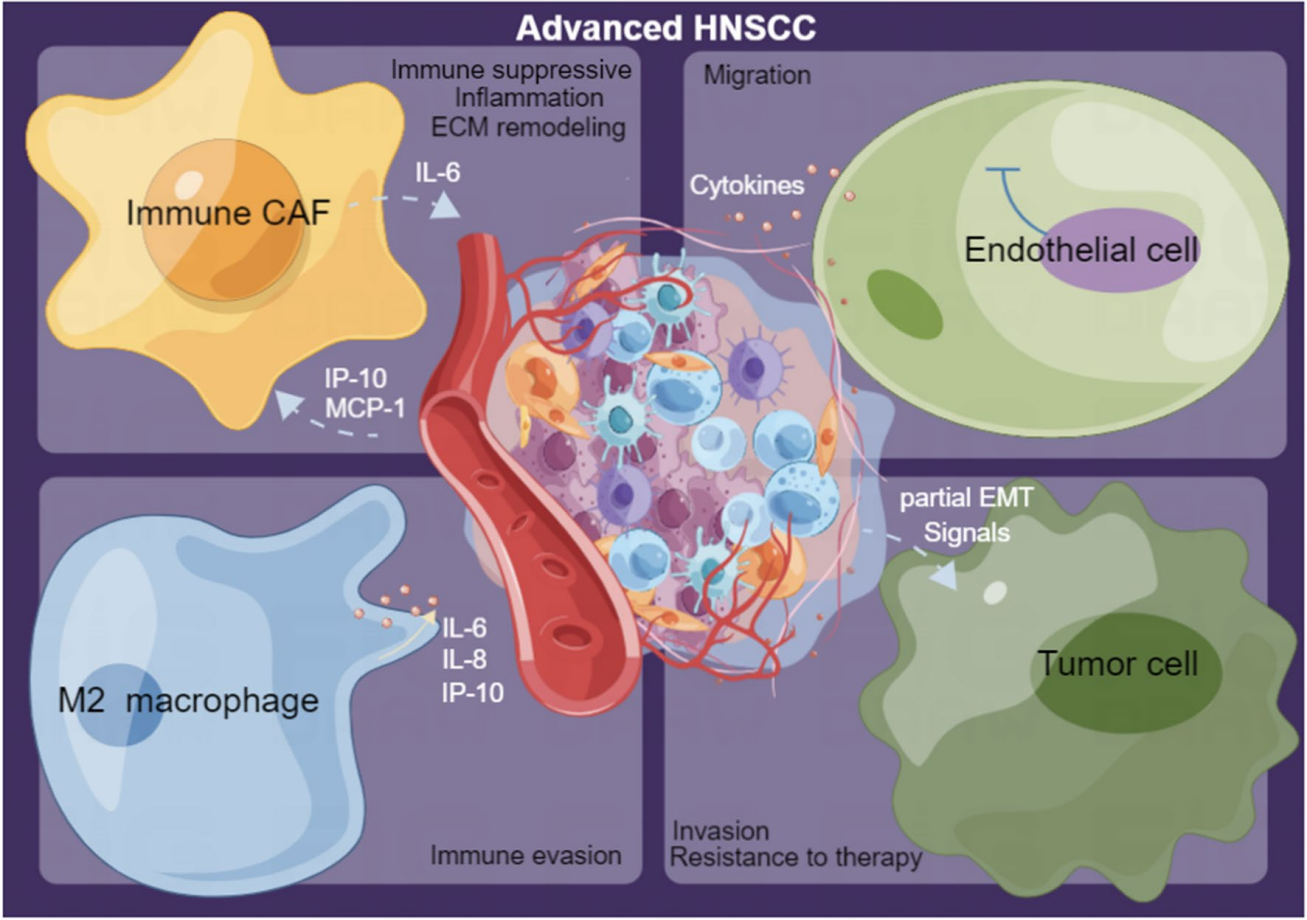

## Introduction

Head and neck cancer is a pervasive malignancy that ranks seventh among all cancers globally. Head and neck squamous cell carcinoma (HNSCC) accounts for approximately 90% of all head and neck cancers and has consistently increased in incidence over the past decade [1]. The burden of HNSCC varies across regions and is often linked to exposure to tobacco carcinogens, excessive alcohol consumption, and infection with oncogenic strains of human papillomavirus (HPV) [2]. Multiple factors, including cancer stage, site, and HPV status, influence the prognosis and survival rates of HNSCC, which are generally poor. Late-stage laryngeal and hypopharyngeal cancer patients have an overall 5-year survival rate of only 40% and 25%, respectively [3, 4]. Despite multimodal treatment being the standard approach for this condition, treatment options remain limited for patients with refractory or metastatic HNSCC. Furthermore, the combination of chemotherapy and radiotherapy frequently leads to severe acute and late toxicities as demonstrated by various studies [5]. Therefore, strategies for treating the disease while minimizing toxicity have been receiving renewed attention. Immunotherapies are emerging as a novel alternative that may provide long-term remission and lower treatment toxicity for patients with refractory or metastatic HNSCC [6].

The tumor microenvironment (TME) in HNSCC is a diverse and intricate combination of tumor cells and stromal cells, including endothelial cells, cancer-associated fibroblasts (CAFs), and immune cells like macrophages [7]. Tumor recurrence and metastasis are strongly influenced by TME [8, 9]. The TME of HNSCC typically exhibits certain characteristics that result in a decrease in anti-tumor immune activity [10], which plays a critical role in regulating the development and progression of HNSCC, and has been identified as a key factor in its metastasis and treatment [7]. Hence, discoveries regarding the regulation of TME in HNSCC hold tremendous therapeutic potential.

There is an urgent need for the development of in vitro models that can accurately replicate tumor-immune interactions in a relevant TME context. Drug candidates identified through human cell line-derived xenografts have poor clinical translation for many cancers, which are not suitable for the development of immunomodulatory drugs due to their significant differences from primary human tumors in terms of proliferative capacity, TME, and compromised immune systems [11]. Immune-competent and genetically engineered mouse models partially overcome these limitations by allowing for a complete immune system, but they still rely on murine stromal and immune components [12]. Cultivating cells in vitro offers the considerable advantage of high controllability, facilitating reproducible experiments at an affordable cost, and thereby leading to widespread applications. Conventionally, two-dimensional (2D) cell culture, where cells grow adherently as a monolayer on the culture vessels, is the most extensively employed technique for in vitro culture. Compared to 2D cultures, three-dimensional (3D) multicellular tumor spheroids are more effective in mimicking the drug response of primary human tumors and have been useful in studying tumor physiology, including metabolic and chemical gradients, hypoxic environments, as well as cell–cell and cell–matrix interactions [13–16]. Drawing from these findings, it is becoming increasingly apparent that the exploration of 3D models could provide more clinically relevant data and could potentially revolutionize our current understanding of cancer biology [17, 18].

In this study, a cell coculture model was established, which encompassed four cellular components: HNSCC cells, human fibroblasts, human monocytes, and human endothelial cells. Compared to the 2D-4-culture model, the 3D-4-culture model exhibited observed migration and transformation of each cell component, as well as changes in cytokine and drug sensitivity.

## Materials and methods

### Cells

The HNSCC cell line FaDu, initially acquired from Suzhou Bei Na Chuanglian Biotechnology (#BNCC316798), was cultured in MEM medium supplemented with 10% (v/v) FBS (all from KeyGEN BioTECH). To facilitate monitoring during co-culture experiments, the FaDu cell line was fluorescently labeled with CellTrace™ CSFE (ThermoFisher Scientific) following the manufacturer's instructions.

Human fibroblasts (HF) were isolated as previously described [19], and cultured in a high-glucose DMEM medium supplemented with 10% (v/v) FBS (all from KeyGEN BioTECH). During co-culture experiments, the HF cell line was fluorescently labeled with CellTrace™ Far Red (ThermoFisher Scientific).

THP-1 cell line was purchased from KeyGEN BioTECH (#KG224), and cultured in RPMI-1640 media supplemented with 10% (v/v) FBS (all from KeyGEN BioTECH). The THP-1 cell line was fluorescently labeled with CellTrace™ Violet (ThermoFisher Scientific) during co-culture experiments.

The HUVEC cell line was obtained from KeyGEN BioTECH(#KG419) and cultured in high-glucose DMEM medium supplemented with 10% (v/v) FBS (all from KeyGEN BioTECH).

Fluorescently labeled cell lines were employed to monitor the different cell types during co-culture. All cultures were incubated at 37 °C in a humidified atmosphere containing 5% CO_2_ and sub-cultured every 2–3 days.

### Co-culture

Mixed cells (FaDu: HF: THP-1 = 2 $\times$ 10^4^:1 $\times$ 10^4^:1 $\times$ 10^4^) were cultured in the conventional 6-well cell culture plate (2D-4-culture) and 6-well SPL3D cell floater plate (3D-4-culture) respectively, with high-glucose DMEM medium supplemented with 10% (v/v) FBS (all from KeyGEN BioTECH). Single HUVECs were seeded in Transwell inserts at a density of 1 × 10^4^ cells, with high-glucose DMEM medium supplemented with 10% (v/v) FBS (all from KeyGEN BioTECH). To quantify the number of migrated HUVECs, images from three different fields of view were captured and analyzed using Image J software (version 1.51j8).

### Confocal microscope

After labeling the cells with fluorescence in 3D-4-culture, the cell spheroids should be transferred gently onto a confocal dish. The three-dimensional cellular dynamics can then be observed using a confocal microscope on days 1, 3, 6, and 9.

### Cell counting kit-8 (CCK-8)

Mixed cells were plated in a traditional 96-well plastic plate and a 96-well SPL3D cell floater plate at a density of 4000 cells/100 µl culture medium. The cells were then treated with varying concentrations of cisplatin. At 24, 48, and 72 h, 10 µl of CCK-8 (Biosharp) was added to the culture medium, and the mixture was incubated at 37 °C for 3 h. The absorbance at 450 nm was measured using a Microplate Reader (PerkinElmer).

### Western blotting (WB)

After cell lysis with RIPA buffer containing PMSF, phosphatase inhibitors, and protease inhibitors, the protein concentration was determined using a BCA protein assay kit. The proteins were then denatured, separated by SDS-PAGE, and transferred onto PVDF membranes. The membranes were blocked with 5% BSA at room temperature for 1 h, incubated overnight with primary antibodies at 4 °C, and subsequently incubated with secondary antibodies at room temperature for 1 h. ECL Western Blotting Detection reagent was applied for chemical development (all from Biosharp).

### Human essential immune response panel (13-plex)

The culture supernatant was centrifuged to remove debris. Next, a determination buffer (25 µL), sample (25 µL), and mixed beads (25 µL) were added to each well. The mixture was oscillated at approximately 500 rpm for 2 h at room temperature in the dark. After two washes, detection antibodies (25 µL) were added to each well and the mixture was oscillated at approximately 500 rpm for 1 h at room temperature. Subsequently, SA-PE (25 µL) was directly added to each well, and the mixture was oscillated at approximately 500 rpm for 30 min at room temperature. Following two washes, the samples were analyzed on a flow cytometer (all from BioLegend).

### Statistical analysis

GraphPad Prism 8.0 software (San Diego, CA, USA) was used to analyze the raw data, which were expressed as mean ± standard deviation (SD). Student's t-test was employed to determine the statistical significance between the two groups. Values of *P < 0.05, **P < 0.01, and ***P < 0.001 were considered statistically significant.

## Results

### Establishment and characteristics of HNSCC co-culture models

**Fig. 1:**
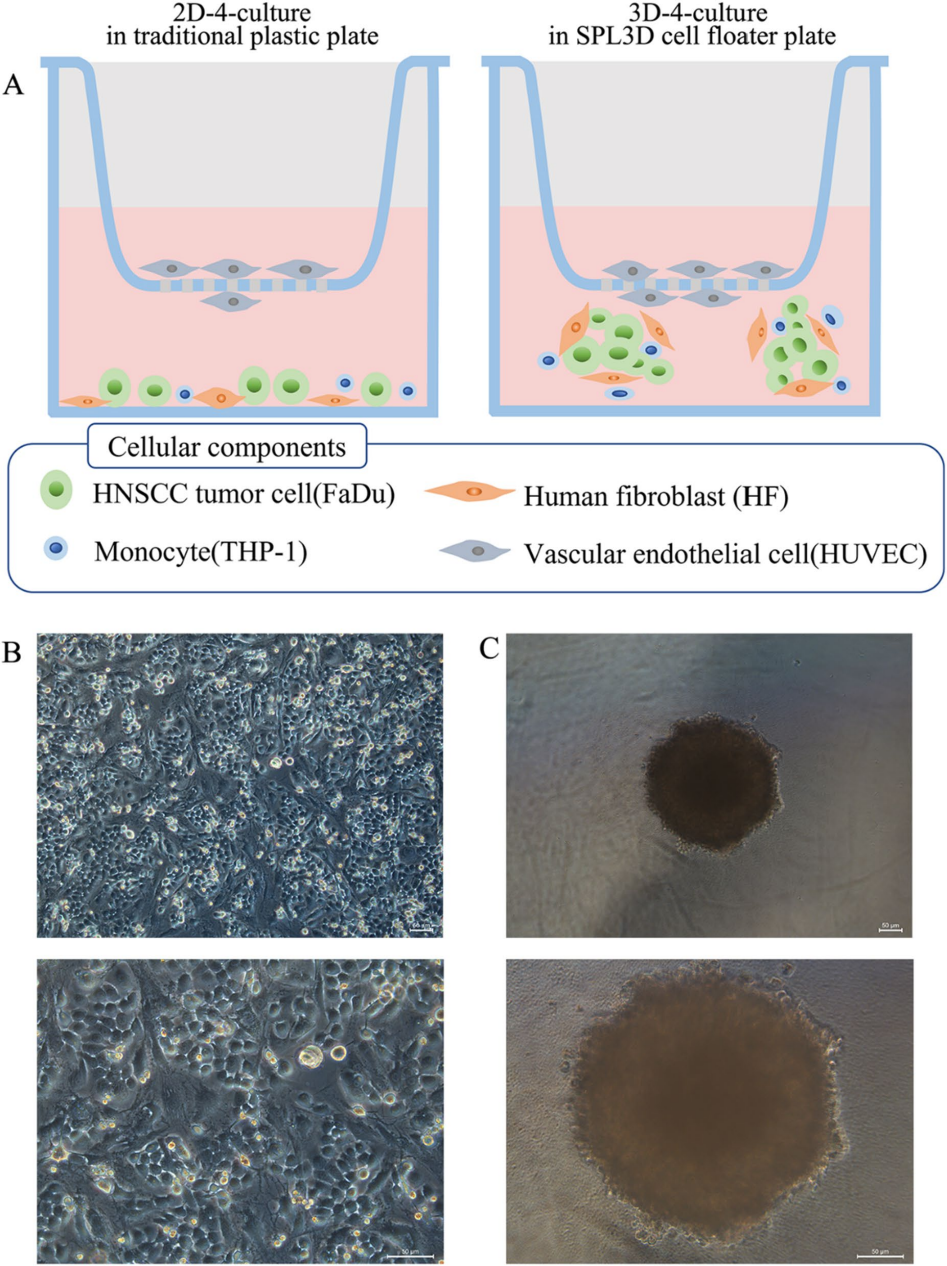
Establishment of HNSCC polyculture models and culture monitoring over time. **A** Schematic of 2D-4-culture and 3D-4-culture: mixed cells (FaDu, HF, and THP-1 cells) were inoculated into conventional cell culture plate and SPL3D cell floater plate respectively, and single HUVEC cells were inoculated into cell culture insert. Representative bright field microscopy images of 2D-4-culture (**B**) and 3D-4-culture (**C**). Scale bar: 50 μm

To establish 2D-4-cultures and 3D-4-cultures, mixed cells (FaDu: HF: THP-1 = 2 $\times$ 10^4^:1 $\times$ 10^4^:1 $\times$ 10^4^) were cultured in a traditional 6-well plastic plate and 6-well SPL3D cell floater plate, respectively. Single HUVECs were then inoculated into cell culture inserts at a seeding density of 1 × 10^4^ cells (Fig. 1A). After a 48-h culture period, the 2D-4-cultures exhibited the formation of typical monolayers (Fig. 1B), while cell spheroids were observed in the 3D-4-cultures (Fig. 1C).

### Live-cell fluorescence imaging of cells stained with live cell trackers via confocal microscopy

**Fig. 2:**
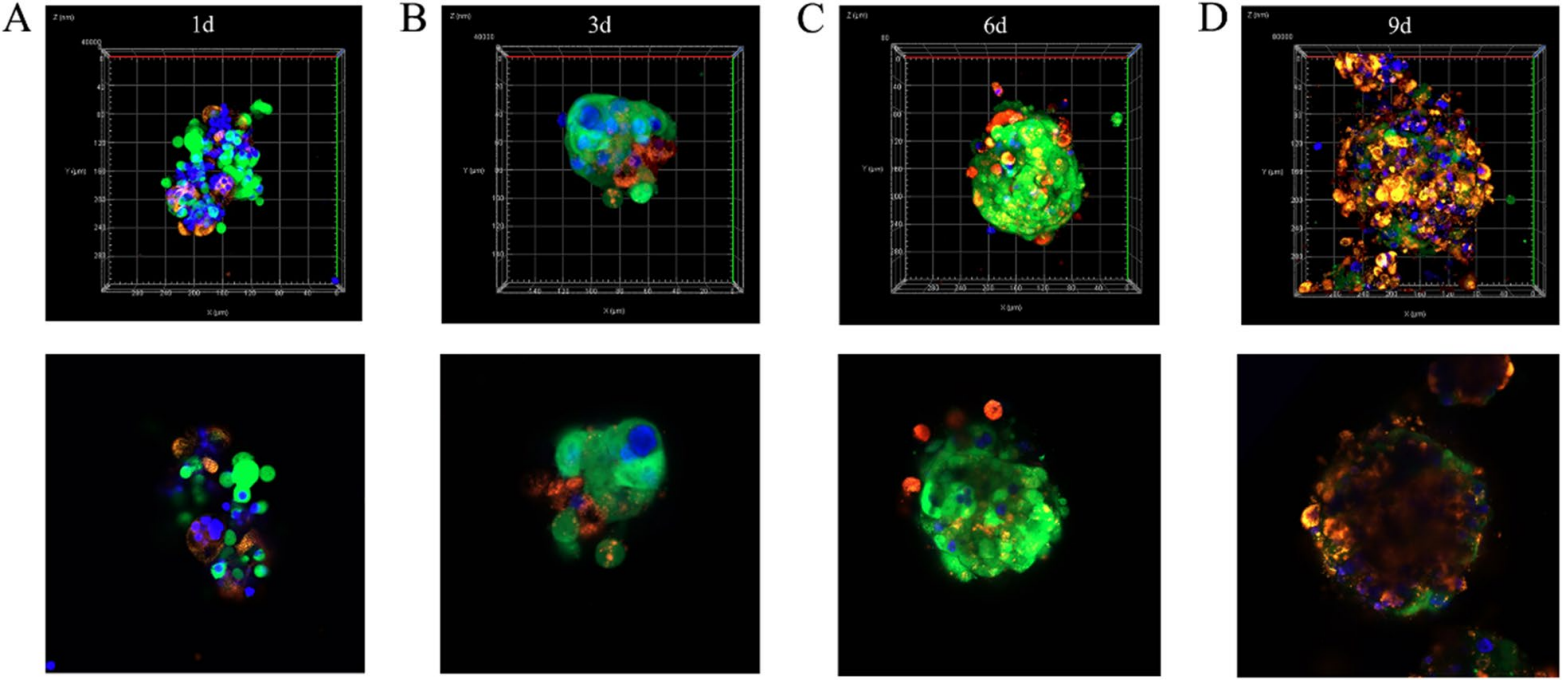
The 1st (**A**), 3rd (**B**), 6th (**C**) and 9th (**D**) day of 3D-4-cultures visualized by confocal microscopy. The cellular types were labeled respectively by CellTrace™ CSFE (green)-FaDu; Cell tracker CM-DiI (red)-HF; Cell tracker CMAC (blue)-THP-1

Rapid 3D growth was observed in the 3D-4-cultures, starting as loose clusters on day 1 (Fig. 2A) and developing into fully formed compact spheroids by day 3 (Fig. 2B). To trace cell movement, cells were labeled with different fluorescent dyes before adding them to the SPL3D cell floater plates. Over time, FaDu cells invaded from the outer layer of spheroids (Fig. 2B, C) and migrated into the interior of spheroids (Fig. 2D), while THP-1 cells were mainly found at the spheroid boundary.

### Cisplatin sensitivity of cells was compared when cells were in 2D-4-culture and 3D-4-culture

**Fig. 3:**
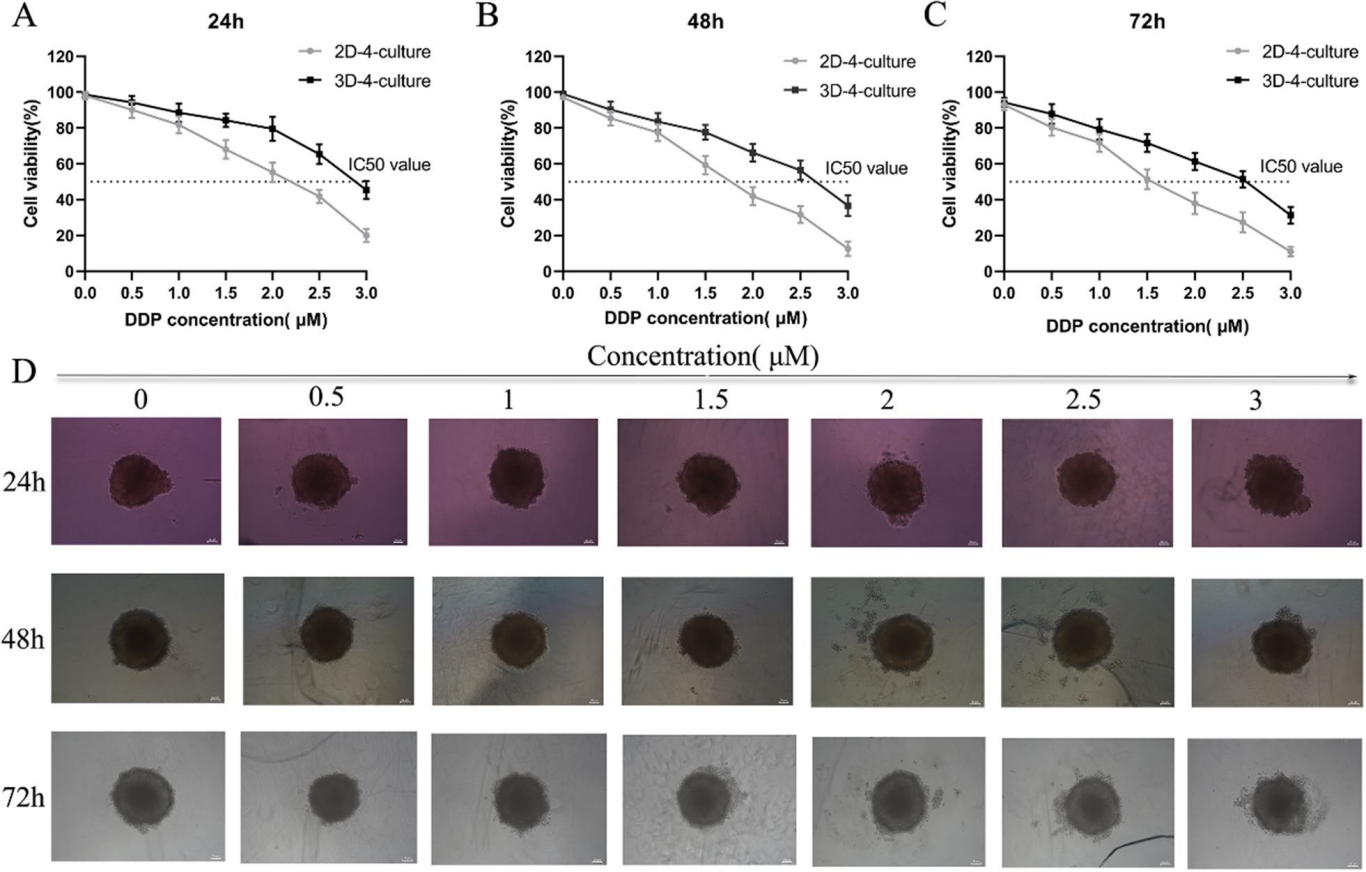
Differences of drug resistance in 2D-4-culture and 3D-4-culture. Cell viability of cells in 2D-4-culture and 3D-4-culture treated with cisplatin by different concentrations for 24 h (**A**), 48 h (**B**), and 72 h (**C**). **D** Representative bright field microscopy images of cells in 3D-4-culture treated with cisplatin by different concentrations for 24 h,48 h, and 72 h. Statistical significance, **P < 0.01, ***P < 0.001

Cells cultured in 2D-4-culture and 3D-4-culture were treated with different concentrations of cisplatin for 24, 48, and 72 h. A significant increase in cisplatin IC50 was observed in cells cultured in 3D-4-culture compared to those in 2D-4-culture: 2.03 ± 0.93 μM vs 3.02 ± 0.87 μM (P = 0.062) on day 1 (Fig. 3A), 1.68 ± 0.94 μM vs 2.62 ± 0.89 μM (P = 0.002) on day 2 (Fig. 3B), and 1.47 ± 0.92 μM vs 2.35 ± 0.88 μM (P = 0.007) on day 3 (Fig. 3C). These findings indicate decreased sensitivity to cisplatin in 3D-4-culture. Additionally, image analysis over time showed lower compactness in 3D spheroids as the concentration of cisplatin increased (Fig. 3D).

### EMT-like phenotype in 2D-4-culture and 3D-4-culture

**Fig. 4:**
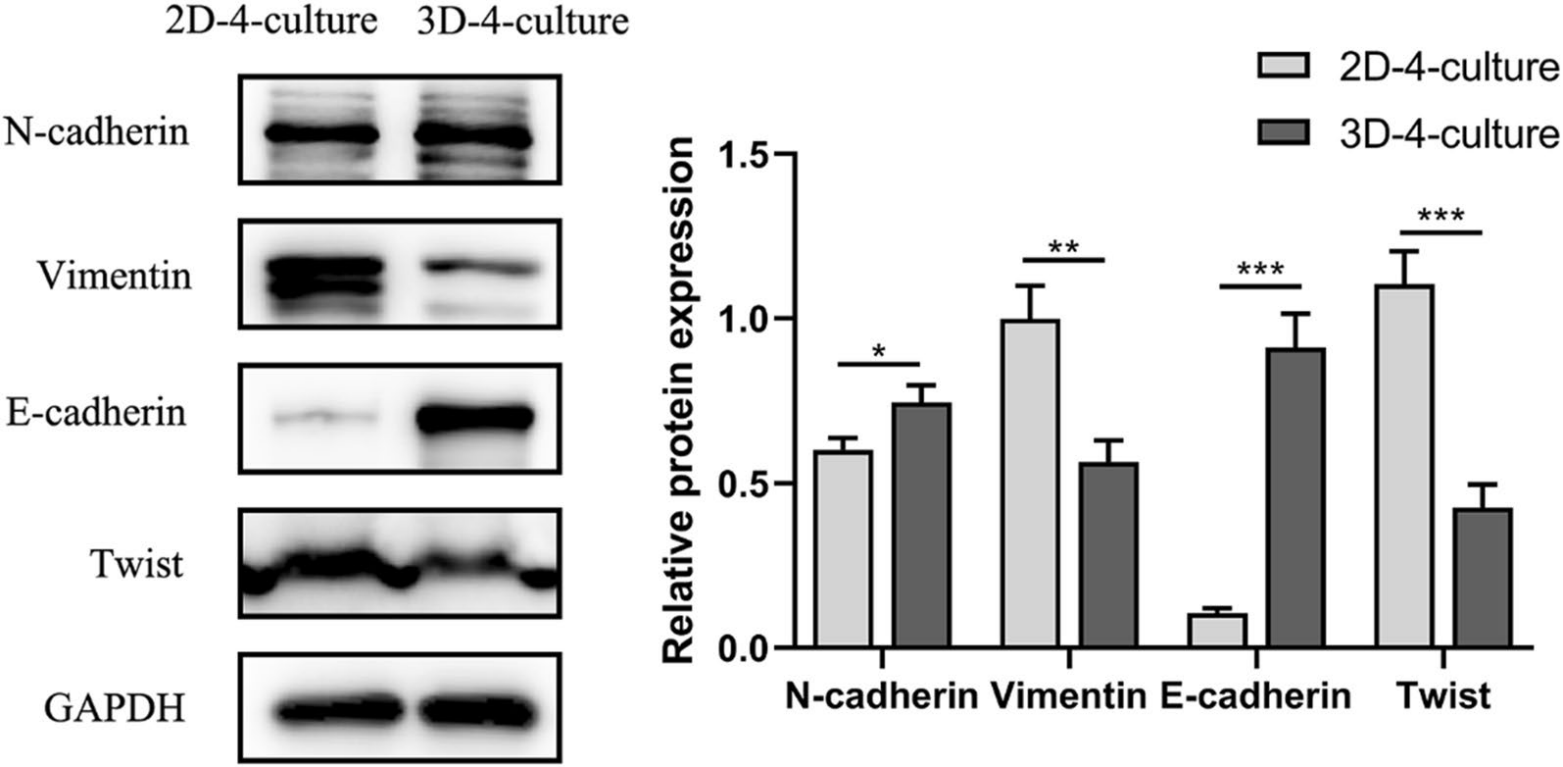
The expression of EMT biomarkers in 2D-4-culture and 3D-4-culture. *P < 0.05, **P < 0.01, ***P < 0.001

After co-culturing for 48 h in 2D-4-culture and 3D-4-culture, the expression of EMT markers was examined. The 3D-4-culture showed a partial EMT phenotype, such as a lack of the mesenchymal marker vimentin and enhanced expression of the mesenchymal marker N-cadherin and epithelial marker E-cadherin, compared to the 2D-4-culture. Moreover, the expression of transcription factor Twist was low, indicating the existence of EMT-like phenotypic changes between the 2D-4-culture and 3D-4-culture (Fig. 4).

### Expression of CD163 and FAP was compared when cells were in 2D-4-culture and 3D-4-culture

**Fig. 5:**
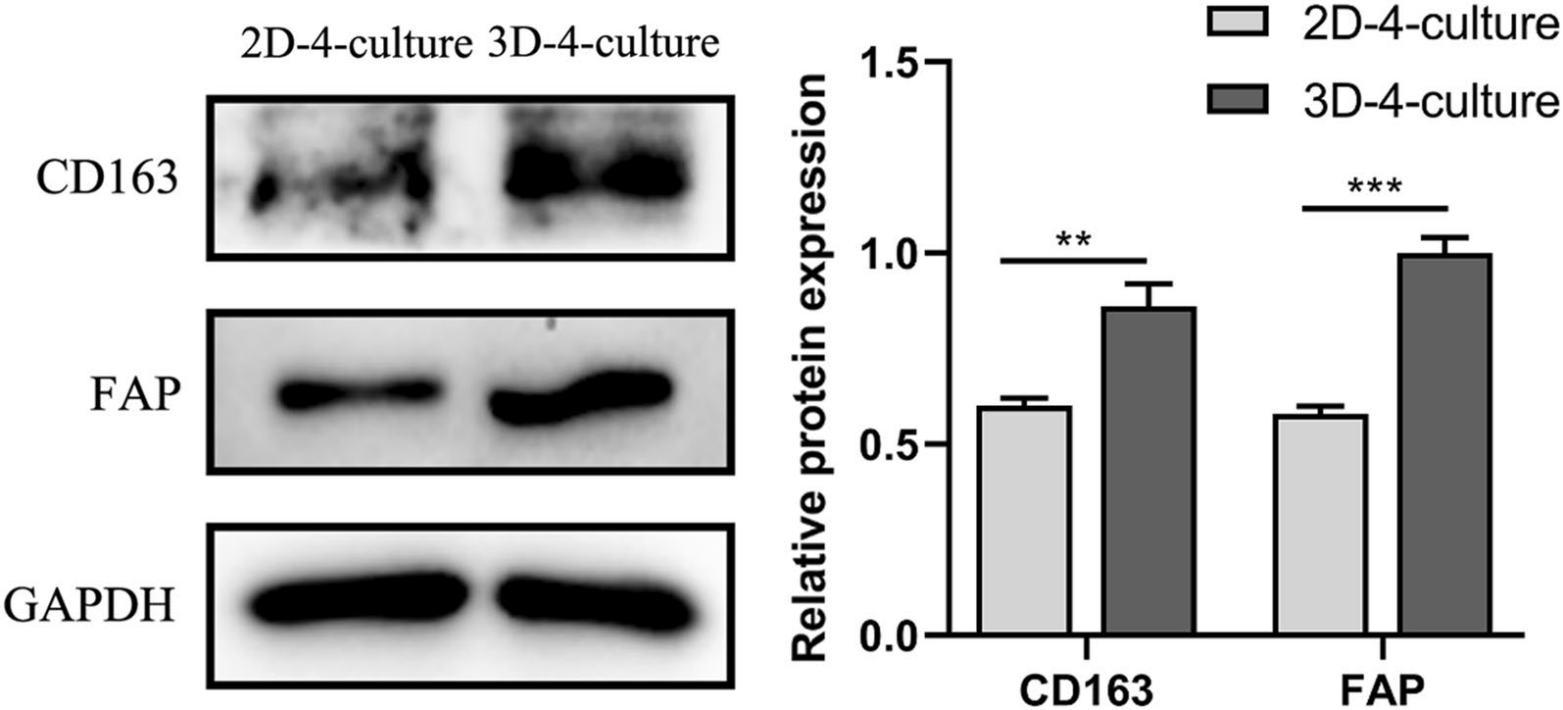
The expression of CD163 and FAP in 2D-4-culture and 3D-4-culture. ***P < 0.001

Cocultured for 48 h, the expression of M2 TAMs marker (CD163) and CAFs marker (FAP) in 3D-4-culture was significantly higher than in 2D-4-culture (Fig. 5).

Following 48 h of co-culture, the expression of M2 TAMs marker CD163 and CAFs marker FAP in 3D-4-culture was significantly higher than that in 2D-4-culture (Fig. 5).

### The chemotactic effect on HUVECs in 2D-4-culture and 3D-4-culture.

**Fig. 6:**
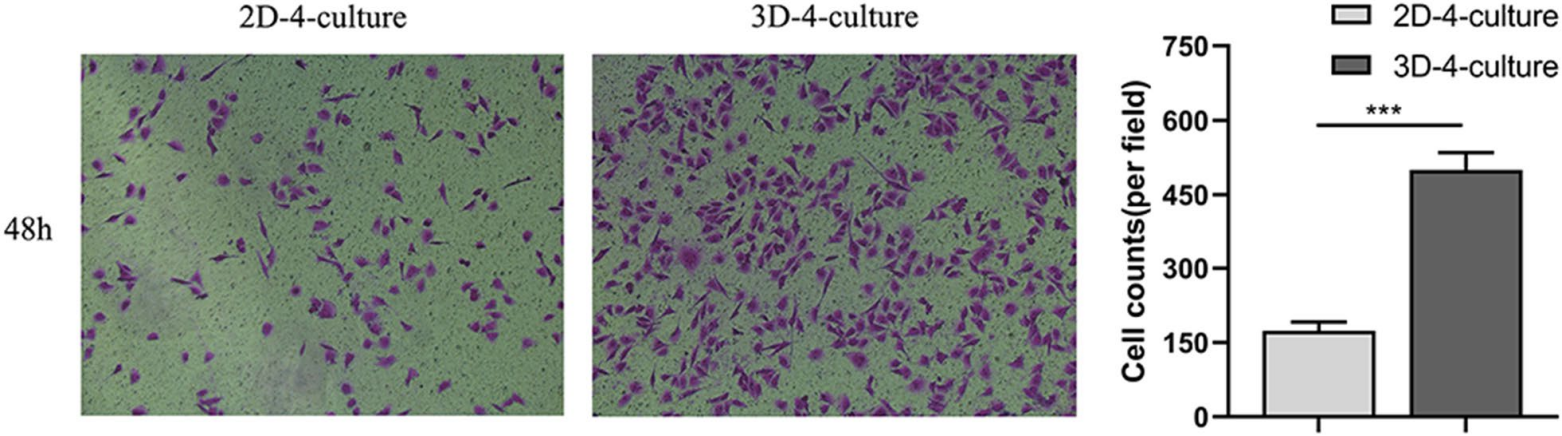
The migrating numbers of HUVECs in 2D-4-culture and 3D-4-culture***P < 0.001

After 48 h of co-culture, the number of HUVECs on the lower surface of the Transwell chamber was 174.00 ± 17.09 in 2D-4-culture and 500.33 ± 35.02 in 3D-4-culture (Fig. 6) (P < 0.001).

### 13 key targets essential for immune response in 2D-4-culture and 3D-4-culture

**Fig. 7:**
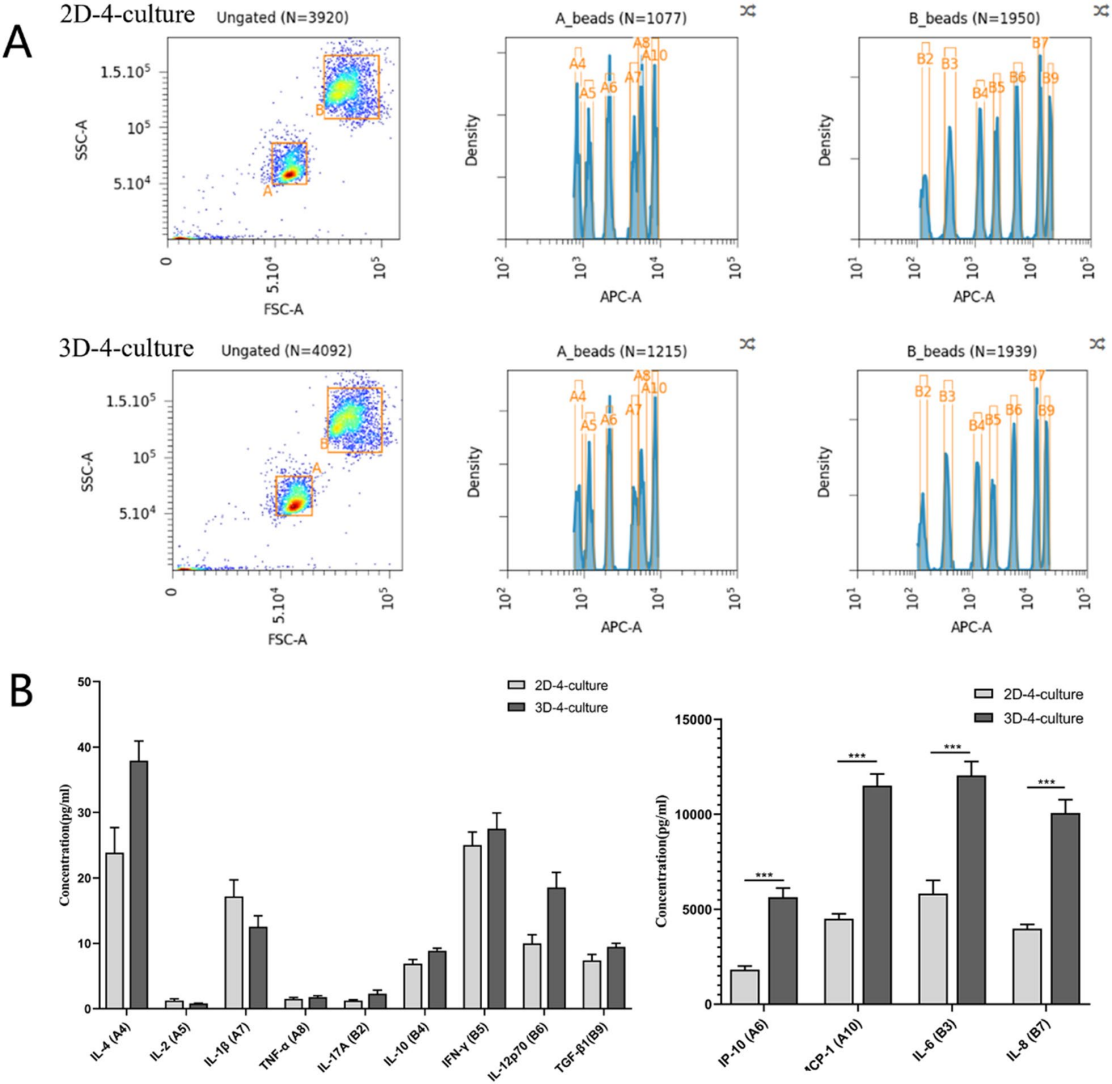
**A** Beads Classification of cell culture supernatant in 2D-4-culture and 3D-4-culture. The smaller Beads A consists of 6 bead populations (A4:IL-4, A5:IL-2, A6:IP-10, A7:IL-1β, A8:TNF-α, A10:MCP-1) and the larger Beads B consists of 7 bead populations (B2:IL-17A, B3:IL-6, B4:IL-10, B5:IFN-γ, B6:IL-12p70, B7:IL-8, B9:Free Active TGF-β1). **B** The concentrations of IL-4, IL-2, CXCL10 (IP-10), IL-1β, TNF-α, CCL2 (MCP-1), IL-17A, IL-6, IL-10, IFN-γ, IL-12p70, CXCL8 (IL-8), and Free Active TGF-β1 of cell culture supernatant in 2D-4-culture and 3D-4-culture. ***P < 0.001

The Beads of the Essential Immune Response Panel possess a unique size that can be identified based on their forward scatter (FSC) and side scatter (SSC) profiles, allowing for the distinction of 13 bead populations differentiated by size and internal fluorescent dye (Fig. 7A). Following a 48-h co-culture, the concentrations of IL-4, IL-2, IL-1β, TNF-α, IL-17A, IL-10, IFN-γ, IL-12p70, TGF-β, IP-10, MCP-1, IL-6, and IL-8 were presented in Table 1. Additionally, the concentrations of IP-10, MCP-1, IL-6, and IL-8 were significantly higher than the other nine targets, as shown on the standard curves (Fig. 7B).

**Table 1:** The concentrations of 13 key targets essential for immune response in 2D-4-culture and 3D-4-culture

Cytokines	2D-4-culture (pg/ml)	3D-4-culture (pg/ml)
IL-4	23.88 ± 3.83	37.92 ± 3.01
IL-2	1.25 ± 0.26	0.81 ± 0.06
IL-1β	17.20 ± 2.50	12.56 ± 1.67
TNF-α	1.52 ± 2.50	1.80 ± 0.20
IL-17A	1.25 ± 0.15	2.29 ± 0.56
IL-10	6.89 ± 0.65	8.89 ± 0.38
IFN-γ	25.02 ± 2.00	27.53 ± 2.40
IL-12p70	10.00 ± 1.30	18.53 ± 2.30
TGF-β1	7.41 ± 0.89	9.48 ± 0.54
IP-10	1818.83 ± 195.96	5635.37 ± 471.29
MCP-1	4501.92 ± 258.42	11,507.70 ± 610.52
IL-6	5821.76 ± 693.03	12,041.87 ± 735.85
IL-8	3986.01 ± 208.70	4501.92 ± 258.42

## Discussion

The crucial role played by the immune system in the initiation, progression, and dissemination of HNSCC has been well-established [8, 9]. Immunotherapy has recently emerged as the most significant therapeutic advancement in the treatment of HNSCC. The introduction of new immune-based therapies for cancer necessitates the use of novel and intricate preclinical models to measure their effectiveness. Conventional treatments targeted the intrinsic growth of cancer cells and could be simulated using 2D monoculture models; however, immunotherapies require significantly greater complexity as they must incorporate immune cell infiltration, tumor cell recognition, and elimination. This complexity must consider various factors such as extracellular matrix composition, architecture, and mechanobiology to model physical and chemical barriers to immune system infiltration. Furthermore, it is necessary to consider interactions between numerous types of cells to attain an accurate representation. Over the years, different culture platforms have been investigated for modeling the tumor immune microenvironment, including 2D culture, microfluidics, bioprinting, spheroids, and organoids [20]. Among these in vitro culture models, 3D spheroids have become the most utilized due to their ease of production and ability to form on a non-adherent surface [21]. Their unique characteristics mainly arise from their layered structure, which comprises an inner necrotic core, a quiescent zone surrounding it, and an outer layer of proliferating cells [22]. In the present study, we introduce a cellular coculture model (3D-4-culture) that includes HNSCC cells, human fibroblasts, human monocytes, and human endothelial cells in an environment allowing for dynamic interaction between each compartment to be recreated, as well as highlight differences observed in 2D-4-culture and 3D-4-culture. Our findings indicate that the 3D-4-culture better simulates the in vivo immune microenvironment of HNSCC compared to the 2D-4-culture.

Live-cell fluorescence imaging of cells stained with live cell trackers was performed using confocal microscopy to evaluate the dynamic changes of different types of cells in 3D-4-culture. After one day of culture, the cells began to form loose clusters, which developed into compact spheroids after three days (Fig. 2A, B). This suggests that SPL3D cell floater plates provide a suitable cell-anchoring scaffold for cells to attach and form a spherical microtumor mass in vitro. As previous studies have revealed, [23, 24] cells were spatially separated, resulting in fibroblasts being localized only in the central region. Our results were consistent with this approximately within the first five days of cultivation (Fig. 2A, B). Interestingly, FaDu cells gradually infiltrated the interior of the spheroids from the outer layer over time (Fig. 2C, D), while THP-1 cells were mainly found at the boundary of the spheroids, similar to invasive human HNSCC tumors infiltrating the surrounding stroma that contains CAFs [25, 26]. In 2D-4-culture, cells exhibit a flattened morphology as they adhere and spread across the surface of the culture dishes. However, this restricts the ability to observe dynamic cellular changes. Therefore, we believe that cell movement observed in both the core and peripheral regions of mixed-cell spheroids closely resembles that which occurs in vivo [2, 27].

A cell viability assay was conducted to evaluate the cytotoxicity of cisplatin treatment in both 2D-4-culture and 3D-4-culture. The results demonstrated that cells grown in 3D-4-culture had higher IC50 values compared to those in 2D-4-culture. These findings align with work done by Styliani et al., in which HNSCC cells grown in three dimensions displayed reduced sensitivity to both cisplatin and cetuximab treatments [28]. 3D spheroid cell culture replicates the cellular heterogeneity inherent in tumors-contrasting starkly with the uniformity typically exhibited by 2D cell cultures [17]. Our observations showed that FaDu cells tended to infiltrate spheroids' interior from the outer layer over time, potentially contributing to changes in the drug sensitivity of the tumor spheroids. Furthermore, it is worth noting that for 3D spheroid structures with diameters exceeding 500 μm, the transport gradients of oxygen, nutrients, and cellular waste are generally limited to 150-200 μm. The proliferating cellular clusters surrounding these 3D spheroids also play a significant role in drug sensitivity [29], which might lead to a decrease in their cisplatin sensitivity. Moreover, 3D cell culturing partially replicates the tumor microenvironment by restoring interactions between cells and between cells and the extracellular matrix (ECM). This process can increase the drug resistance of tumor spheroids [30, 31]. Spheroids in 3D-4-culture showed lower cell aggregation with increasing concentration of cisplatin, indicating that the density of spheroids was correlated with the activity of the cells.

The transformation of normal cells into malignant cells involves a complex interplay with various cell types present in the tumor microenvironment. Once tumor cells invade the stroma, they can enter circulation and spread to distant organs, a process influenced by the pre-metastatic niche and organotropism. It is becoming evident that the epithelial-mesenchymal transition (EMT), characterized by the loss of epithelial properties and acquisition of mesenchymal traits, plays a crucial role in both primary tumor formation and metastasis [32]. Conversely, mesenchymal-epithelial transition (MET) involves the reversal of EMT and is also observed during tumor metastasis [33]. Certain tumors are capable of colonizing distant locations via MET [34], while E-cadherin expression at a metastatic site may serve as evidence of MET [35]. In contrast with some previous studies that showed spheroid cultures displaying mesenchymal-like traits such as decreased E-cadherin expression and increased N-cadherin and vimentin expression [36, 37], our results demonstrated a partial EMT state in 3D-4-culture, with lower Twist and vimentin expression and higher E-cadherin and N-cadherin expression compared to 2D-4-culture. Similar research has empirically demonstrated that when subjected to 3D culture conditions, there is a notable augmentation in the presence of the cell adhesion molecule E-cadherin in both epithelial breast carcinoma MCF-7 cells and colon adenocarcinoma Lovo cells. This elevation was found to escalate the chemo-resistance towards therapeutic agents such as cisplatin, 5-fluorouracil, and Adriamycin [30]. The consistency between these findings and our results suggests that alterations in EMT levels within the 3D spheroid may also contribute to chemotherapeutic resistance compared to 2D culture.

Initial reports suggest that spheroids are a suitable model for investigating tumor-immune interactions [38, 39], as the architecture of a tumor is critical in determining the immunosuppressive tumor microenvironment found in human tumors [40–42]. Compared to 2D-4-culture, a greater proportion of CD163 + and FAP + cells were exhibited in 3D-4-culture, similar to what occurs with tumor infiltration in HNSCC [27, 43] as well as a notable increase in the passage of HUVECs through compartments. This indicates that the 3D-4-culture model was better suited for replicating key features of the immune microenvironment in HNSCC and was more conducive for cell migration, which is consistent with previous findings in primary HNSCC [2, 44].

The modulation of TAMs and CAFs in co-culture models is primarily influenced by the accumulation of a mixture of soluble factors and observed cell–cell interactions. It is important to note that cytokine supplementation alone did not induce macrophage polarization. Our findings demonstrate that the accumulation of soluble factors, such as IP-10, MCP-1, IL-6, and IL-8, is significantly higher in the 3D-4-culture compared to the 2D-4-culture. Monocyte chemoattractant protein-1 (MCP-1), also referred to as Chemokine (CC-motif) ligand2 (CCL2), can be derived from tumor cells and stromal cells, thereby influencing the regulation of monocytes, macrophages, and T lymphocytes to promote immune suppression [45]. Previous studies have demonstrated that MCP-1 is capable of modulating the tumor progression of oral squamous cell carcinoma by regulating tumor-associated fibroblasts [46]. Interferon-γ-inducible protein 10 (IP-10), also known as Chemokine C-X-C ligand 10 (CXCL10), is predominantly secreted by monocytes, endothelial cells, fibroblasts, and cancer cells. This chemokine has been demonstrated to play a role in hypoxia-induced inflammation [47]. Its immune regulatory function primarily occurs through its interaction with the chemokine receptor CXCR3, predominantly expressed on monocytes and macrophages [48]. Interleukin-6 (IL-6), a cytokine produced by various cell types including immune cells, endothelial cells, and tumor cells, is known to strongly induce epithelial to EMT in HNSCC [49]. It is associated with cancer recurrence, poor prognosis, drug resistance, and tumor metastasis in HNSCC [50, 51]. The tumorigenic effects of IL-6 involve the conversion of tumor-associated macrophages from an M1-type phenotype with anti-tumor properties to an immunosuppressive M2-type phenotype [52]. In addition to its effects on immune cells associated with the tumor, considerable attention has been given to the impact of IL-6 on cancer-associated fibroblasts due to their promotion of tumor growth [53]. Interleukin-8 (IL-8), a cytokine can be secreted by macrophages and endothelial cells, while its corresponding receptors are expressed in granulocytes, monocytes, and endothelial cells [54]. Conditioned media derived from tumor cells encouraged monocyte differentiation into TAMs with an M2-like phenotype. These TAMs secrete elevated levels of IL-8, which in turn regulate several pro-tumor effects [55]. This indicates that the 3D-4-culture model better simulates the immune microenvironment of HNSCC compared to 2D-4-culture.

Furthermore, a clearer understanding of the development of an immunosuppressive environment than 2D-4-culture was revealed through analysis of the secretory profile in the 3D-4-culture model. This may explain the observed induction of TAMs and CAFs phenotypes, which are primarily associated with the accumulation of specific cytokines such as IL4, IL13, IL10, and CXCL1, as previously reported [56–58].

Our study has several limitations. Firstly, HUVECs were cultured in transwell chambers, which only allow paracrine effects on other cells without direct cell-to-cell interactions. Secondly, the issue of reconstructing the complex tumor-associated vascular system in vitro was not adequately addressed. Additionally, further investigation is needed to elucidate the reasons for the differences in cisplatin resistance between 2 and 3D models. Moreover, the underlying mechanisms of partial EMT in vitro remain unclear. Lastly, it remains unclear which cells play a major role in the secretion of cytokines.

In conclusion, we have developed a 3D-4-culture model that mimics the dynamic interactions of the immune microenvironment, accumulation of secreted factors, and impact on chemosensitivity. This contributes to the maintenance of key features in late-stage HNSCC, including chemoresistance. The model allowed for the activation of monocytes into a TAM-associated phenotype without the addition of external factors, as well as the conversion of fibroblasts to CAFs. As such, it is highly suitable for replicating the entire tumor and evaluating individual tumor responses to conventional treatments or immunotherapies.

## Data Availability

The datasets used and analyzed during the current study are available from the corresponding author upon reasonable request.
